# CHALLENGING AGEISM IN HEALTHCARE THROUGH INTERPROFESSIONAL EDUCATION

**DOI:** 10.1093/geroni/igac059.2764

**Published:** 2022-12-20

**Authors:** Min Kyoung Park, Diane Martin

**Affiliations:** University of Maryland, Baltimore County, Ellicott City, Maryland, United States; University of Maryland, Baltimore , Baltimore, Maryland, United States

## Abstract

Broadly defined as prejudice, discrimination, or stereotyping of older adults, ageism is prevalent in the healthcare system. It is implicated in the over- or under-diagnosis and/or treatments provided to older persons, resulting in decreased quality of life and increased costs of care. In Fall 2021, we developed a virtual interprofessional education (IPE) activity to increase professional health and social care trainees’ awareness of ageism and to biases they may hold. During the asynchronous portion of the training, participants complete the Harvard Implicit Association Test (age), watch a video, and respond to related threads on Blackboard, which provides an opportunity to discuss experiences prior to participating in a synchronous training activity incorporating a presentation on ageism in healthcare. To date, 98 trainees have provided qualitative data on the impact of the ageism training components. Overwhelmingly, trainees indicated the value of information provided; many of whom responded that the test revealed their own biases. Participants commented that the training was an opportunity to open their eyes to ageism and that it made them more aware of the importance of understanding older adults’ capabilities and limitations in a respectful way. More than half (55.86%) of threaded discussions noted that the ageism training helped them to realize their biases against older adults and understand older people’s challenges. Quantitative data from pre/post IPE surveys indicated increased interest in working with older adults. Responses illustrate the need to and value of incorporating anti-ageism content into educational programs of our future health and social care workforce.

